# Silk Materials Functionalized via Genetic Engineering for Biomedical Applications

**DOI:** 10.3390/ma10121417

**Published:** 2017-12-12

**Authors:** Tomasz Deptuch, Hanna Dams-Kozlowska

**Affiliations:** 1 Department of Cancer Immunology, Chair of Medical Biotechnology, Poznan University of Medical Sciences, 61-688 Poznan, Poland; to.deptuch@gmail.com; 2 Department of Diagnostics and Cancer Immunology, Greater Poland Cancer Centre, 61-688 Poznan, Poland

**Keywords:** bioengineered silk, biomaterials, functionalization, genetic engineering, biomedicine

## Abstract

The great mechanical properties, biocompatibility and biodegradability of silk-based materials make them applicable to the biomedical field. Genetic engineering enables the construction of synthetic equivalents of natural silks. Knowledge about the relationship between the structure and function of silk proteins enables the design of bioengineered silks that can serve as the foundation of new biomaterials. Furthermore, in order to better address the needs of modern biomedicine, genetic engineering can be used to obtain silk-based materials with new functionalities. Sequences encoding new peptides or domains can be added to the sequences encoding the silk proteins. The expression of one cDNA fragment indicates that each silk molecule is related to a functional fragment. This review summarizes the proposed genetic functionalization of silk-based materials that can be potentially useful for biomedical applications.

## 1. Introduction

Natural silk fibers offer extremely high tensile strength and extensibility while possessing low weight [1]. A new generation of materials based on this natural polymer may have many potential applications. Bullet-proof vests, versatile ropes, parachutes, and surface coatings are a few examples of the possible ways to utilize silk-based materials [2,3,4,5,6]. Because of its exceptional biocompatibility and biodegradability, silk has recently attracted considerable attention in terms of possible biomedical applications. Both natural and recombinant silk can be processed into various morphological forms, such as films, sponges, non-woven mats, hydrogels, scaffolds, capsules or spheres [2,7]. Silk has been proven to be a useful biomaterial for the construction of matrices for tissue engineering and as carrier system of drugs, nucleic acids and proteins [2,3,5,7,8,9].

Natural silk can be harvested from different organisms, but spiders and silkworms are the most common sources of silk. The cannibalistic and territorial behavior of spiders have made it impossible to breed them and thus to obtain sufficient quantities of silk for the production of materials. Moreover, some species of spiders can produce up to seven different forms of silk, which vary in terms of their composition and physical properties [10]. Therefore, the material produced from harvested spider silk would be made from different types of silk proteins, which in turn, would contribute to inconsistencies in the material between batches. Although it is possible to harvest dragline silk as a pure material, it is a relatively inefficient process, and the quality of the silk would depend on conditions such as diet, temperature, and humidity [11].

Another approach to obtaining silk is the processing of silkworm cocoons. Silkworm cocoons are composed mainly of two fibroin proteins coated with highly adhesive sericins, which are responsible for the stability of the cocoon structure [12]. Sericins can be extracted from cocoons during a thermochemical process called degumming [13]. Removing sericins is crucial because the combination of silk fibroin and sericins can induce immunological responses [14]. Although, the regeneration process is relatively cheap and efficient, materials made of regenerated silkworm silks often require further modification and processing in order to gain suitable properties as biomaterial [15,16]. Many of aforementioned drawbacks can be overcome by using the tools of biotechnology. Genetic engineering enables the construction of synthetic genes that encode bioengineered silk. The biotechnological production of bioengineered silks can resolve the problem of silk accessibility. Moreover, genetic engineering can be used to modify or to functionalize the silk biomaterial by manipulation of the amino acid composition or by addition of a fragment that determines a function. Such modifications can expand the already excellent properties of silk and provide an opportunity to further customize silk materials for more tailored uses.

**Figure 1:**
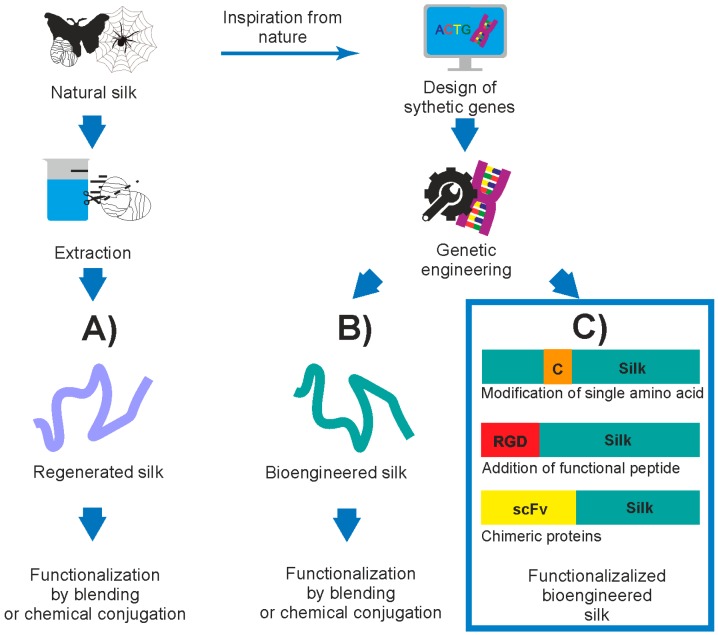
Strategies for acquiring and possibly functionalization of silk proteins. (**A**) Silk can be obtained directly from nature and, after the extraction, can be functionalized by blending with other compounds or by chemical conjugation; (**B**) knowledge of the interplay among the sequence-structure properties of silk proteins enables the design of synthetic genes, and then the bioengineered silk is produced in the heterologous host. The bioengineered silk can be further functionalized by blending with other compounds or by chemical conjugation; (**C**) genetic engineering enables the direct functionalization of silk. The silk sequence can be modified at the DNA level so that, after production in the heterologous host, a functionalized bioengineered silk is obtained. The modifications can refer to addition/substitution of a single amino acid or to fusion of a peptide or a fragment (domain) of another protein. This review covers the functionalization of silk through genetic engineering.

Figure 1 summarizes the current strategies of acquiring and functionalizing silk proteins. Independent of the origin of the silk (directly from nature as a regenerated silk or indirectly from a biotechnological process as a recombinant silk), it can be further processed in order to gain a new function/property. These modifications can be introduced by blending with other compounds or by chemical conjugation, but these types of modifications are not in the scope of the present article and are not reviewed. This review covers the functionalization of silk biomaterials through genetic engineering and their potential application in the biomedical field.

## 2. Silk Proteins and Their Recombinant Variants

Among the spider silk proteins (spidroins), the most commonly investigated for biomedical application, derive from two representatives of the order Araneae: *Nephila clavipes* and *Araneus diadematus* [17]. The proteins major ampullate spidroin 1 (MaSp1) and major ampullate spidroin 2 (MaSp2) from *N. clavipes* as well as *Araneus diadematus* fibroin 4 (ADF4) and *Araneus diadematus* fibroin 3 (ADF3) from *A. diadematus* form the representative dragline silks [11]. These proteins are composed mainly of repeating domains consisting mostly of glycine and alanine [18,19]. Alanine-rich motifs (A)_n_ or (GA)_n_ form β-sheet structures, which are responsible for the strength of silk fibers. The glycine-rich motifs form an amorphous matrix, which provides the elasticity of the fiber. Two glycine-containing motifs GGX form 3_1_–helices, and a proline-containing motif GPGXX is involved in a β-turn spiral formation [18]. The repeated blocks are flanked by non-repetitive and conservative N- and C-termini, which control the solubility of silk and the process of dragline silk formation [17,20,21]. Another well-characterized silk is the silkworm silk derived from cocoons of *Bombyx mori*. Silkworms produce silk fiber consisting of two fibroin proteins: light chain (~26 kDa) and heavy chain (~390 kDa). The light and heavy chains are connected through a disulfide bond. Moreover, the glycoprotein P25 is non-covalently attached to the heavy–light chain complex in a ratio of 1:6:6 (P25:heavy chain:light chain, respectively). The core of the heavy chain is made of the repeating motifs of (GAGAGS)_n_ that form the β-sheet structures contributing to the strength of silk fiber [22].

As mentioned above, although silk fibroin is accessible from *B. mori* cocoons, spidroins are mainly produced using biotechnological processes. The expression of recombinant silk proteins by using naturally originating cDNA in bacteria was not effective [23]. The large size of silk constructs and their repetitive character led to translation errors, protein accumulation in inclusion bodies and ultimately very low protein yields [23,24,25]. The most efficient strategy for expressing recombinant silk proteins is based on the synthetic genes composed of the desired number of repeating nucleic acid blocks that encode the silk multimers. Based on the amino acid sequence of the natural silk proteins, the short monomeric modules of the synthetic gene can be designed and then synthesized [26]. A larger, multimeric structure can be obtained as a result of concatemerization, step-by-step ligation or recursive directional ligation [26,27]. Designed genes encoding bioengineered silk proteins can be expressed in different hosts. The production of genetically engineered (bioengineered) silk proteins has been performed in yeast (*Pichia pastoris*), insects (silkworm larvae), plants (soybean, *Arabidopsis*, potato, tobacco), mammalian cell lines (Baby hamster kidney (BHK) cell line) and transgenic animals (mice, goat) [28,29,30,31,32,33]. The most commonly used expression system for the production of recombinant silk proteins is a bacterial system. The simplicity of the production, the short generation time of bacteria, the low cost and the ability to scale up the process are the main arguments supporting the use of this system [27].

Although the synthetic gene design and the recombinant production of silk generate a great opportunity to obtain silk of desirable properties, this method still needs some improvements. The main drawbacks of the production of recombinant silk are the possible endotoxin contamination, low yield of particular silk protein variant and the scale-up of the process. The size and highly repetitive sequences encoding silk proteins can limit the expression yield in the heterologous host. The stock of desirable nucleotides, tRNAs, RNA polymerases, and ribosomes in the host needs to be evaluated for the demands of the silk expression. An example of the engineering of the host’s metabolic pathways to increase a yield and quality of the recombinantly produced silk has been indicated [34]. Moreover, recent progress in metabolomics, synthetic/systems biology, mathematical and computational modeling may help to overcome the obstacles of recombinant silk production [35].

Aside from silkworms and spiders, other animals like bees, ants, and hornets also produce silks. Unlike silkworm and spider silks, the silks from these species are not large, repetitive fibrous proteins but consist of four small, non-repetitive coiled coil proteins and the genes encoding these proteins have been identified [36,37]. Due to their size and structure, the coiled coil silks may be better suited to the recombinant production in the heterologous host. Indeed, the efficient production of this type of silk has been reported [38,39]. Moreover, the recombinantly obtained silks self-assembled to reproduce the native coiled coil structure and were able to form fibers [39]. Furthermore, the fibers assembled from a single recombinant silk protein were structurally and mechanically similar as natural silk formed from four types of coiled coil proteins [40]. The simplicity of their transgenic production and unique structural properties make coiled coil silks very promising proteins for developing a new class of materials for various biomedical applications.

Knowledge about silk’s structure (the structural motifs that compose the building blocks) and corresponding functions enables the design of new materials mimicking the properties of their natural equivalents [41]. This approach offers an ability to control the properties of the biomaterial precisely. Changes in the molecular weight and amino acid sequence of the protein produced and the position of building blocks can affect silk properties such as the secondary structure content, solubility, hydrophobicity and charge [42]. These, in turn, will affect the morphology of the final material [42]. Moreover, a recent report indicated that the methodology of the recombinant silk purification also affected the morphology and property of the final silk biomaterial [43]. Knowledge of these correlations may allow the design de novo of silk-based biomaterials. Furthermore, computational modeling may help to generate materials with tunable properties [44]. Mesoscopic dissipative particle dynamics (DPD) simulation was used to develop a coarse-grained description of silk proteins as multiblock copolymers. Then the model was applied to reveal the processing conditions and design parameters that control silk fiber assembly [44]. The model was validated by experimental data [44]. Simulation programs may help to reduce the time required for designing and characterize new materials based on silk. This can be of great importance for the development of materials for biomedical applications and beyond. The interplay among the sequence-structure properties of silk-based proteins and also the use of computational modeling have been reviewed previously [17,45].

Aside from direct control over material properties, genetic engineering enables the functionalization of silk materials. The nucleic acid sequences that encode the peptides or fragments (domains) of other proteins can be fused to the silk sequences. These added amino acid sequences impart new, desirable characteristic to the silk polymer. Genetic engineering can be a useful tool to adopt and expand the application of silk in the biomedical field and beyond.

## 3. Functionalization of Silk by Changing Its Amino Acid Sequence

The functionality of silk-based materials can be achieved by modification of the amino acid sequence of silk protein. Addition or substitution of a single amino acid possessing a side chain with a particular property and located in a strategic position in the protein sequence can provide additional control over the properties of silk-based materials. Flanking the polyalanine region of a recombinant 6-mer silk protein (based on MaSp1 spidroin from *N. clavipes*) with methionine allowed effective control over the solubility of silk [46]. Methionine acted as redox trigger. In the reduced state, β-sheet structures were formed, while selective oxidation of methionine residues resulted in inhibition of β-sheet formation, increased bulkiness and increased hydrophilicity of silk [46]. Additional control over solubility can greatly improve the purification efficiency of silk by preventing its premature precipitation. Moreover, such modification can be utilized to design stimuli-responsive materials that can transition from the aqueous to solid state in response to a stimulus. Furthermore, silk films with lower crystallinity bound positively charged drugs more efficiently than did more crystalline films [47,48]. Through manipulation of the secondary structure of silk, it can be possible to control the drug binding and release profile. Moreover, the content of the secondary structure of silk can also affect its biodegradability [49,50]. After exposure to proteases, silk fibroin hydrogels showed different (faster) degradation profiles than films with highly crystalline structures [50].

Modification of the amino acid sequence can result in alteration of the overall charge of recombinant silk. Substitution of negatively charged amino acids with cationic ones can greatly improve the cellular uptake of silk-based carriers. Spheres formed from a cationic variant (eADF4(_K_16)) of the recombinant silk protein eADF4(C16) (based on ADF4 spidroin from *A. diadematus*), in which all glutamic residues were substituted with lysine, displayed a significant increase in the internalization rate compared with the unmodified eADF4(C16) spheres [51].

The insertion of cysteine into the amino acid sequence of eADF4(C16) silk introduced a new binding site for molecules [52]. The thiol group present on the cysteine residue could form covalent bonds with various compounds, such as dyes, biotin or β-galactosidase [52]. Moreover, such a modification potentially allows the formation of multilayer and multipurpose materials based on such silk.

The functionalization by modification of amino acid sequence may not only relate to the repetitive region of silk but also to its terminal domains. The double mutant of the N-terminal domain of MaSp1 silk derived from *Euprosthenops australis* was generated and then used for recombinant protein production and purification purposes [53]. By replacing aspartic acid 40 with lysine and lysine 65 with aspartic acid at the N-termini, a mutated variant was generated and named NT*. The NT* variant was pH insensitive, stabilized and hypersoluble compared to the wild-type equivalent. Its application enabled the recombinant production of various hydrophobic and/or aggregation-prone proteins with much higher yields as compared to the application of other commonly used solubility tags [53]. This novel solubility enhancing fusion tag may allow a recombinant production of different proteins that are pharmaceutically relevant and can be applied in the clinic.

## 4. Functionalization of Silk by Addition of Functional Peptides

Functional peptides are composed of several amino acids (usually 8–12) and possess a certain capability, such as anti-microbial activity, enzymatic activity, receptor ligand recognition or binding of a target molecule. By using genetic engineering methods, the nucleic acid sequences encoding these peptides can be introduced into the coding sequence of bioengineered silks. This simple genetic modification has enabled the formation of functionalized silk materials suitable for drug delivery and tissue engineering. The functional peptides described in this review are summarized in the Table 1.

### 4.1. Functionalization of Silk for Cellular Targeting of the Drug Delivery Systems

Drug delivery systems should help to minimize the adverse effects of therapeutics by reducing the undesirable interactions between the drug and organism. Encapsulation of a therapeutic into a vehicle should not only diminish its toxicity but also protect the therapeutic from degradation upon systemic administration. Another attribute of delivery systems should be the selective targeting of a particular organ, tissue or cell before releasing the loaded drug. Moreover, the vehicles for drug delivery should also be non-toxic and biodegradable. Unmodified silk-based drug delivery systems address only some of these aspects. Although there are known examples of silk carriers that are non-toxic and biodegradable and can effectively protect their cargo from degradation [54,55], these unmodified silk carriers did not allow for targeted delivery and showed negligible cellular uptake [54,56]. Proposed anti-cancer therapies utilizing unmodified silk fibroin carriers for the delivery of small drugs such as doxorubicin (Dox) [57] paclitaxel (PTX) [58], curcumin [59] or cisplatin [60] assumed that the deposition of carriers would occur at the tumor site due to the enhanced permeability and retention (EPR) effect. The leaky vascular system of tumor tissue and impaired lymphatic drainage allows accumulation of small particles and carriers at the tumor site [61].

The internalization of silk-based carriers into cells can be improved by the introduction of the proper functional peptide into the bioengineered silk. The addition of poly-lysine (KKK KKKKKKKKKKKK) or poly-arginine blocks (RRRRRRRR) significantly increased the uptake of silk spheres into cells [51,55,62]. The positive charge of these residues also allowed for the binding of nucleic acids through interactions with the negatively charged phosphate backbone of nucleic acids [55,62]. The presence of such peptides did not impair the ability of MS2 (based on MaSp2 spidroin from *N. clavipes*) and 6-mer (based on MaSp1 spidroin from *N. clavipes*) silk to self-assemble, and these functionalized silks were able to form spheres or polyplexes with nucleic acids [55,62]. The 6-mer silk fused with positively charged peptides could be utilized as a silk-based film for the local delivery of therapeutic genes [62]. Silk-based gene delivery systems can be a good alternative to the currently available viral vectors, which might have adverse effects after in vivo application [63].

To further improve internalization into cells, the integrin binding motif or various cell-penetrating peptides (CPPs) have been applied in bioengineered silk carriers [9,51,54,64,65,66]. Integrin-binding motifs composed of the amino acid sequence RGD are recognized by integrins–transmembrane proteins that are involved in cell-extracellular matrix (ECM) attachment. The introduction of the RGD motif into bioengineered 6-mer silk carriers increased their internalization into cells [64]. The number of RGD motifs was crucial; the silk carriers with the highest number of RGD repeats (11x) had the highest transfection efficiency [64]. Moreover, the addition of CPPs allowed for better cellular internalization of modified silk spheres. These short (mostly approximately 30 amino acids in length) peptides have a positive charge, which facilitates crossing through the cell membrane. Their sequence can be designed, such as peptides based on a poly-arginine (R) n motif, or they may derive from proteins existing in nature. One of the most characterized CPPs is a peptide derived from the transactivator of transcription (Tat) protein of human immunodeficiency virus (HIV) [67]. The fusion of the Tat peptide (RKKRRQRRR) to the silk sequence improved the uptake of eADF4(C16)Tat silk-based variants into HeLa cells compared to the unmodified variant [51]. The fusion of lysine-rich, cell membrane-destabilizing peptides ppTG1 (GLFKALLKLLKSLWKLLLKA) also enhanced the cellular uptake of silk-based gene carriers (complexes) into HeLa cells [54]. ppTG1 alone provided a high transfection rate but did not protect the cargo (nucleic acids) from enzymatic degradation [68]. In contrast, the carriers based om 6-mer silk containing the ppTG1 and poly-lysine peptides enabled both binding and protection from degradation of nucleic acids [54]. Furthermore, the formed ppTG1 silk/plasmid DNA (pDNA) complexes exhibited a sustainable and controlled release of pDNA by enzymes that degrade silk protein [54].

Although the CPPs facilitate cellular internalization, they lack cell specificity. To achieve greater selectivity, the peptides that recognize the particular molecule on the cell surface can be fused to silk. Tumor-homing peptides (THPs) efficiently target the tumor microenvironment [69]. The F3 peptide (KDEPQRRSARLSAKPAPPKPEPKPKKAPAKK) binds specifically to nucleoin (the molecule expressed on the surface of angiogenic endothelial cells and some tumor cells), and the CGKRK peptide to heparan sulfate present in tumor vessels [70]. Both peptides were successfully fused to a 6-mer protein functionalized with a poly-lysine peptide for nucleic acid binding [66]. The CGKRK peptide and F3 functionalized silk/pDNA complexes showed target specificity towards MDA-MB-435 melanoma cancer cells and highly metastatic human breast tumor MDA-MB-231 cells but not to the control, non-tumorigenic MCF10A cells (mammary breast epithelial cells) [66]. The transfection rates of both constructs were significantly higher in the tested cancer cells than in the control cells in the in vitro studies [66]. Numata et al. formed nanocomplexes of pDNA and a bioengineered silk monomer (1-mer) (based on MaSp1 spidroin from *N. clavipes*) that was functionalized with a poly-lysine peptide for nucleic acid binding and with F3 or Lyp1 peptides [65]. The Lyp1 peptide (CGNKRTRGC) targets the lymphatic vessels of certain types of tumors [71]. The nanocomplexes of such functionalized silk/pDNA also showed higher transfection rates in MDA-MB-231 and MDA-MB-435 cells compared with the unmodified variant and with non-tumorigenic mammary breast epithelial cells [65].

Modification of silk proteins that target human epidermal growth factor receptor 2 (Her2) was also proposed [56,72]. Her2 is overexpressed in 20–30% of invasive breast carcinomas [73]. Spheres made of recombinant MS1 protein (based on MaSp1 spidroin from the *N. clavipes* spider) functionalized with Her2 binding peptides showed binding and cellular uptake in Her2-positive SKOV3 and SKBR3 cell lines, contrary to that observed for plain MS1 spheres and Her2-negative control cells [56]. Two variants of tumor-homing peptide were evaluated—H2.1 (MYWGDSHWLQYWYE) and H2.2 (LTVSPWY)—at different configurations (fusion at the N and C termini). Variants with H2.1 or H2.2 binding peptides on the N-termini showed higher binding efficiency than did the C-termini variants. Moreover, these peptides for targeted delivery did not impair the ability of silk proteins to self-assemble and, therefore, spheres were successfully produced. Furthermore, the functionalized spheres loaded with the model drug doxorubicin (Dox) were able to transport the cytotoxic drug into target cells effectively [56]. The same peptides (H2.1 or H2.2) were used to functionalize MS2 silk, however the binding of these spheres to target cells was substantially lower compared with the binding of the spheres composed of functionalized MS1 equivalents [72]. Thus, a new approach was applied by blending the two functionalized silk types at different weight ratios. The spheres formed by the blending of functionalized MS1 and MS2 silks at a ratio of 8:2 were bound to the target cells at the same level as functionalized MS1 spheres but had greatly improved physical-chemical properties. Moreover, compared with functionalized MS1 spheres, functionalized MS1:MS2 particles efficiently killed targeted cells when loaded with a drug inducing considerably lower non-specific toxicity [72].

An alternative strategy for silk-based drug delivery systems involves the use of other tumor-specific ligands, for example, nucleic acid sequences such as CpG-siRNA [55]. Spheres made of MS2 functionalized with the poly-lysine peptide KN (KKKKKKKKKKKKKKK) showed efficient loading of CpG-siRNA. These silk spheres protected their cargo from degradation in serum and enabled the internalization into target cells and the sustainable release of an siRNA-based therapeutic into the cytoplasm [55]. Moreover, the encapsulation into bioengineered silk spheres changed the kinetics of CpG-STAT3siRNA processing, resulting in delayed and prolonged silencing of the target molecule (in this case, the signal transducer and activator of transcription 3 (STAT3)) compared with the control (naked CpG-STAT3siRNA) [55]. In this strategy, the target specificity was provided by the sequence of the nucleic acid CpG, which selectively affected the cells expressing Toll-like receptor 9 (TLR9). However, the proposed system is versatile and can be used for loading different nucleic acid-based therapeutics (targeting different cells or silencing different genes), expanding possible targets of silk-based nucleic acid delivery systems.

### 4.2. Functionalization of Silk for Cell Adhesion

The biocompatibility of silk-based materials and their unique physical properties render them useful in the field of tissue engineering. The functionalization of silk materials can help to better address the needs of regenerative medicine. The addition of integrin-binding motifs (like RGD) not only mediated the internalization of silk-based drug carriers into cells [51,64] but also promoted cell adhesion and proliferation on silk-based matrices [74,75,76,77]. Cell adhesion molecules such as integrins allow cells to adhere to the ECM through ligand-receptor interactions [78]. The RGD motif binds to a wide range of integrins [78]. The addition of RGD to 15-mer silk-based films promoted the adherence and proliferation of human mesenchymal stem cells (hMSCs) [74]. Mouse fibroblasts (BALB/3T3) showed greater adhesion to eADF(C16) silk films functionalized with the RGD peptide than did unmodified silk films [77]. Moreover, bioengineered silk functionalized with RGD was more prominent than silk that was chemically modified with the RGD motif in terms of cell adhesion and proliferation [77]. The advantages of using genetically functionalized silk are that the production is easier and faster than the chemical coupling reaction, which requires additional steps and is not entirely effective [77]. Moreover, the chemically modified silk also poses a problem of possible contamination and further side effects in humans.

Widhe et al. analyzed fibroblasts, keratinocytes, endothelial cells and Schwann cells in the terms of their adhesion to matrices formed from bioengineered 4RepCT silk (based on MaSp1 spidroin from *E. australis*) functionalized with integrin-binding peptides e.g., peptides of amino acid sequences RGD or IKVAV [75]. The cells showed higher adhesion to matrices (fibers, films and foams) produced from silk functionalized with RGD than to silk matrices without such a modification. Schwan cells also displayed better adhesion to films containing the IKVAV peptide than to unmodified 4RepCT matrices [75]. These functionalized materials could be used as scaffolds for cell culture studies or as materials for tissue regeneration. Binding peptides (peptides of amino acid sequences: RGD, IKVAV and YIGSR) were also fused to 4RepCT bioengineered silk and used as a scaffold for in vitro pancreatic cell culture [76]. YIGSR is a fragment of domain III of the laminin β1 chain and a major cell receptor binding site in laminin. These modifications (especially with the RGD peptide) enhanced the ability of the silk scaffold to maintain a human pancreatic islet culture and allowed for higher cell proliferation [76]. This system can be applied for diabetes treatment. Because of the large number of viable islets that is required for effective transplantation, the most efficient approach could be the isolation of pancreatic islets and their in vitro culturing before engraftment. Functionalized silk scaffolds can help in obtaining a sufficient number of islets for effective diabetes therapy.

The YIGSR and RGD motifs were also successfully introduced into the silk fibroin sequence through transformation of *B. mori* preblastodermal stage eggs [79]. DNA vectors were used to transform the silk’s heavy or light chain with either the YIGSR motif (TS(CDPGYIGSRAS)_8_) or the YR motif composed of YIGSR (TS(CDPGYIGSRAS)_8_) and RGD ((TGRGDSPAS)_8_). As a result, a transgenic silkworm strain capable of producing functionalized silk was obtained [79]. Cells showed significantly better adhesion to the film containing only the TS(CDPGYIGSRAS)_8_ motif, independent of the location (L- or H-chains), than to the native films. Moreover, an in vivo study indicated that materials made of silk containing the laminin peptide YIGSR are promising in terms of application as biocompatible vascular grafts [79].

The length, number, surrounding amino acids and secondary structure of peptides containing the RGD motif play significant roles in the cell-adhesion process [80]. The specific integrins possess an affinity towards defined domains incorporating the RGD motif [81]. The secondary structure of the RGD motif used for silk functionalization may, therefore, have a significant role in the process of cell attachment. Among three different forms of peptides containing the RGD motif (one cyclic form and two linear control forms) fused to 4RepCT silk, the cyclic form provided the best adhesion of keratinocytes to the silk matrices (films) [80]. Cyclization of the peptide was achieved through insertion of cysteine residues into its sequence (CTGRGDSPAC) [80].

Silk functionalized for better cell adhesion can be utilized as a coating for implantation materials. The silk coatings can help attach the surrounding cells to the implant surface, which may in turn reduce the convalescence time and increase the biocompatibility of material used for implantation (e.g., titanium). Unmodified 4RepCt silk and bioengineered 4RepCt silks functionalized with the cell-binding peptide from fibronectin (CTGRGDSPAC) and with the anti-microbial motif Mag (GIGKFLHSAGKFGKAFVGEIMKS) were successfully processed into coatings for titanium, stainless steel, hydroxyapatite, and polystyrene matrices [6]. The coating process did not influence the functionality of the bioengineered silk. The better cell adhesion to Fn-functionalized silk and the improved anti-microbial properties of Magfunctionalized silk demonstrated that such bioengineered silks can be applied as coating materials for inorganic implants [6].

### 4.3. Functionalization of Silk with Anti-Microbial Properties

Biomaterials with anti-microbial properties for use in tissue engineering would help prevent local infections at the implantation site, significantly reducing patient convalescence time. In addition to the anti-microbial silk 4RepCT functionalized with the Mag peptide described above [6], bioengineered silks have been functionalized with other peptides possessing anti-microbial properties, such as human neutrophil defensin 2 (HNP-2) (CYCRIPACIAGERRYTSGTCIYQGRLWAFCC), human neutrophil defensin 4 (HNP-4) (VCSCRLVFCRRTELRVTSGNCCLIGGVSFTYCCTRV) and hepcidin (DTHFPICIFCCGCCHRSKCGMCCKT), and examined to evaluate their suitability as anti-microbial materials [82]. The soluble bioengineered 6-mer silk proteins functionalized with HNP-2, HNP-4 or hepcidin showed microbicidal activity towards gram-negative *Escherichia coli* and gram-positive *Staphylococcus aureus* strains when tested in the radial diffusion assay [82]. Furthermore, these modified silks preserved the self-assembly property and formed films that were not cytotoxic towards human osteosarcoma cell lines (SaOs-2) [82]. Further in vivo research showed that silk-based films containing the hepcidin domain triggered a mild to low immunological response, similar to that observed in control groups (silk 6-mer without hepcidin, polylactic-glycolic acid (PLGA) films and animals that had undergone the same surgical procedure but without implantation of material) [83]. The fusion of silk and antimicrobial peptides may represent a promising approach to the generation of materials that prevent infection after their implantation. In addition to the potential applications in biomedicine (as mentioned above, e.g., vascular grafts, coatings for implants, and matrices for tissue engineering), silk with anti-microbial activity may find utility in the cosmetics and food industries.

### 4.4. Functionalization of Silk for Binding Inorganic Molecules

Organisms such as diatoms (*Cylindrotheca fusiformis*) are capable of producing versatile 3D porous structures made of silica under mild physiological conditions. Silaffin proteins play a major role in this process. The R5 peptide (SSKKSGSYSGSKGSKRRIL) derived from silaffin was able to bind and deposit silica in a controlled fashion [84]. To gain control over biosilica formation, the R5 peptide was fused to bioengineered silks [85,86,87]. The fusion of a 15-mer protein to the R5 peptide generated nanocomposite materials containing silica particles with a narrow size distribution of 0.5–2 µm in diameter. Moreover, this hybrid silk was able to form films and fibers [85]. Film based on a recombinant silk 6-mer protein functionalized with the R5 peptide was reported to not only deposit biosilica on its surface but also control the size and rate of silica mineralization through control of the β-sheet content of the silk protein [86]. The modification of silk with the R5 peptide was the most promising functionalization compared with other modifications such as fusion to peptides A1 (SGSKGSKRRIL) and A3 (MSPHPHPRHHHT) [86]. Furthermore, the 15-mer silk protein functionalized with the R5 peptide formed a composite silk/silica film that promoted the proliferation of hMSCs and their differentiation into an osteogenic lineage, which was indicated by the presence of osteogenic gene markers [87]. These properties render R5-functionalized silk matrices useful for bone-regeneration purposes. 

Another approach to generate silk-based materials suitable for bone reconstruction involves fusion of the peptide named VTK (VTKHLNQISQSY) with a 15-mer silk protein [88]. This peptide, discovered using a phage-display technique, effectively bound hydroxyapatite (HA) and had a critical role in the induction of biomineralization [88]. The VTK peptide was fused on the N- or C-terminal or on both terminals of the bioengineered silk protein, and the functionalization on both terminals was the most effective at increasing the formation of crystalline hydroxyapatite. Films made of silk-VTK bioengineered proteins were non-toxic, and they not only successfully induced biomineralization but also enhanced hMSC differentiation compared with the control material made of unmodified silk [88].

Silk-based materials have also been successfully modified to bind metals. Functionalization with peptides that bind silver nanoparticles enabled the design of silk-based films displaying anti-microbial properties [89]. Fusion of 12-amino-acid peptides Ag-4 (NPSSLFRYLPSD) and Ag-P35 (WSWRSPTPHVVT), identified by the phage-display technique, with 6-mer and 15-mer bioengineered silk proteins induced the formation of silver nanoparticles from silver nitrate solution (AgNO_3_) [89]. Moreover, this functionalization did not impair the self-assembly of silk. The films made of such functionalized silk proteins, after deposition of silver nanoparticles, offered prolonged anti-microbial activity towards gram-positive and gram-negative bacteria [89]. As mentioned above, anti-microbial activity is a very desirable property for materials used for implantation and regenerative medicine.

Uranyl ions could be bound to silk after functionalization with U1 (EQIAEFKEAFALCTKDGTGYITTKELGTCMRSLTS) and U2 (EQIAEFKEAFALCTKDGTGYITTKELGTCMRSLTS)_2_ peptides derived from a mutated fragment of calmodulin from *Paramecium tetraurelia* [90]. The point mutations made in the native peptide, which was responsible for calcium binding, allowed selective binding to uranyl ions [91]. These peptides were used to modify a 6-mer silk protein, which resulted in obtaining a functional fusion protein capable of chelating uranyl ions [90]. Such modified silk can be applied to design new biomaterials useful for the treatment of human exposure to uranium, for the bioremediation of contaminated environments, or for the construction of biosensors.

## 5. Functionalization of Silk by Designing Chimeric Proteins

Knowledge of the relationship between the structure and function of protein building blocks enables the design of new biomaterials inspired by proteins occurring in nature. Moreover, fusion of the sequences derived from different proteins allows the generation of biomaterials with unique biophysical and biochemical properties that are precisely tailored to the application. Silk chimeras generated by this strategy can benefit from silk’s strength, biocompatibility and ability to self-assemble into higher structures, while the used motif/domain of the other protein incorporated into the silk provides a function. Table 2 summarizes chimeric silk-based proteins.

### 5.1. Chimeric Biopolymers

An example of such a hybrid construct is the silk elastin-like protein (SELP) [92,93,94,95,96,97,98]. SELPs are based on tandemly repeated units of the *B. mori* silk motif (GAGAGS) and the mammalian elastin sequence motif (GXGVP, where X can be any amino acid except proline) [96]. SELPs are composed of semi-crystalline (silk) blocks and elastomeric (elastin) blocks. The silk peptides self-assemble into insoluble β-sheet structures to provide thermal, chemical and mechanical stability, while elastin undergoes a reversible structural transition to provide the dynamic function [96]. At an appropriate concentration and temperature, SELPs can transition from a soluble form into hydrogels via rapid hydrophilic-to-hydrophobic transitions in response to stimuli such as temperature, pH and ionic strength changes [96,97]. The number of repeating blocks and the X residue in the elastin sequence determine the physicochemical properties of the material [94,98]. Because of its soluble state at a certain temperature and solid state at body temperature, this material can act as a solvent-free and injectable depot for tissue-localized drug and gene delivery and tissue-regeneration purposes [92,93,95,99]. Silk-elastin chimeras have been reviewed in detail elsewhere [96].

Collagens, the major structural proteins of the ECM, play significant roles in cell signaling, differentiation and development [100]. Collagens obtained from animals are commonly used in regenerative medicine and tissue engineering [101]. To ensure greater homogeneity and safety (transmission of diseases), a few recombinant variants of collagen have been proposed [102]. A collagen-like protein derived from *Streptococcus pyogenes* possesses properties similar to eukaryotic collagen [103]. The bacterial collagen trimerization domain (V) and collagen-like domain (CL) were fused to a repetitive *B. mori* silk consensus sequence (GAGAGS)_n_, then expressed in bacteria and purified [104]. Other variants were generated that contained the integrin and fibronectin binding sequences (GFPGER and GLPGLAGQRGIVGLPGQRGER, respectively), which were introduced into the collagen domain [104]. These silk chimeric proteins were successfully bound to previously prepared solid silk fibroin matrices (films and scaffolds). As a result, material mimicking the ECM environment was generated due to the presence of integrin and fibronectin binding motifs in the collagen-like domain [104]. These binding sites induced faster proliferation of hMSC cells compared with the cells grown on the control matrices. The fusion of silk and collagen proteins generated a functional material, where the silk sequence provided controllable, specific binding to the crystalline repetitive domain of the silk biomaterial while the collagen-like domain that contained integrin- and fibronectin-binding sites interacted with the cell surface and promoted cell growth [104].

Silk–collagen chimeras composed of hydrophobic collagen-inspired and histidine-rich silk-inspired blocks could also provide a robust platform for stimuli-responsive hydrogels [105]. Due to the presence of the collagen domains at the flanking positions of the silk blocks, the chimeric protein transformed from a soluble form into solid fibers and hydrogels at physiological pH [105]. However, due to the lack of cell-binding motifs in this silk–collagen chimera, cells did not spread and attach as well as cells in the control (collagen) group; this silk–collagen hydrogel supported rat bone MSC growth in osteogenic medium [105]. The main advantage of the silk–collagen chimera was the absence of observed contraction of the material during the 21 days of cell culture. Contraction of the material is a major drawback of collagen-based gels because the tightening of the hydrogel network may lead to ejection of Ca from the material [105].

### 5.2. Silk Chimeric Proteins for Binding Inorganic Molecules

The fusion of the 156-amino-acid carboxyl terminal domain of dentin matrix protein 1 (CDMP1) with repeating blocks of a 15-mer spider silk protein generated a new functional material [106]. DMP1 possesses the ability to nucleate hydroxyapatite and is expressed in osteoblasts, osteocytes or ameloblasts. Through nucleation of HA, DMP1 influences the bone-reconstruction process. Durable films made of this chimeric protein were formed, and they nucleated HA [106]. These properties make silk–dentin chimeric films promising materials for bone and dental tissue engineering.

Bioengineered silk based on a 6-mer and bone sialoprotein (BSP) chimeric protein was used to produce thin films mimicking the ECM for bone tissue regeneration [107]. BSP is expressed by osteoblasts (especially in mineralized connective tissue) and promotes cell adhesion and differentiation, playing an important role in bone formation and remodeling [108]. In vitro studies showed that a silk–BSP chimeric protein induced the nucleation of calcium phosphates (CaP) [107]. The silk–BSP chimeric films induced hMSCs differentiation into osteoblasts. During two weeks of culturing of hMSCs on the silk–BSP film, the cells were capable of sustaining proliferation and differentiation into the osteogenic linage in an osteogenic medium [107]. Moreover, further in vivo evaluation in mice indicated that silk–BSP films did not show major differences in inflammatory response compared with the control [109].

### 5.3. Silk Chimeric Proteins for Binding Organic Molecules

The ability to bind various organic molecules can notably increase the relevance of silk as a biomaterial. Fragments of proteins, such as the albumin-binding domain (ABD), biotin-binding domain (M4), IgG-binding domain (C2) from streptococcal protein G (SPG) and IgG-binding domain (Z) from staphylococcal protein A (SPA), were successfully fused to 4RepCT bioengineered silk without hindering the silk’s ability to self-assemble into fibers and films [110]. Furthermore, fusion and processing into matrices did not impair the ability of the added domains to selectively bind the target molecules. Moreover, the matrices that were made of two different silk fusion proteins displayed combined binding properties. The proposed method of functionalization of the material (by fusion) is of great importance since many molecules often lose their bioactivity upon immobilization. The ability to bind specific molecules can be utilized in different types of assays in which silk can act as an immobilization platform [110]. Moreover, the ability to bind organic molecules such as biotin may allow for generating multi-layer materials composed of differently functionalized materials linked through biotin/streptavidin interaction.

Thatikonnda et al. proposed a silk-based material composed of silk fused with a single-chain variable fragment (scFv) derived from a human antibody [111]. The scFv combines variable fragments of light and heavy antibody chains—the parts responsible for antigen recognition. Several variants of silk chimeras were produced via genetic fusion of two scFvs that recognized different serum proteins (i.e., VEGF (vascular endothelial growth factor) and C1q (complement component 1, q subcomponent)) and two partial silk variants named RC and NC (based on MaSp1 spidroin from *Euprosthenops australis*). RC silk was composed of a repetitive part and C-terminal domain of spidroin, and the NC variant was generated by fusion of the N- and C-termini of spidroin (without a repetitive fragment). In these chimeras, the antigen recognition by the scFv domains was preserved, and these hybrid proteins were able to bind their targets selectively. Interestingly, the fusion of scFv domains did not impair silks processing into fibers, especially taking into consideration the similar size of the fused components. Moreover, the scFv/NC variants indicated better solubility properties that allowed nanodispensing of these silks into nanoarrays [111]. This kind of material appears to be a promising candidate for next-generation immunoassays and biosensors.

Another useful functionalization of silk-based materials for use in diagnostics and biosensors is the fusion of silk with enzymes [112]. Silk can act as a robust frame, whereas the functional part of the enzyme will provide a catalytic functionality for the bioengineered material. Successful fusion of xylanase with 4RepCT silk indicated that such modifications were indeed possible and effective [112]. The chimeric protein maintained its enzymatic properties under continuous substrate flow. It was durable during cleaning with ethanol and could be reused and stored [112]. Furthermore, these properties were preserved after processing the chimeric protein into different morphological forms (fibers, films and foams) [112].

Recombinant silk based on a 15-mer protein fused with a cellulose-binding domain (CBD) could efficiently bind cellulose nanocrystals (CNCs), resulting in the formation of a silk–CBD–CNC nanocomposite [113]. Like silk, cellulose possesses outstanding mechanical properties and is often used in the formation of transparent films and hydrogels. However, its application in vivo is rather limited due to the harsh environment required for its processing. The presence of a CBD domain causes silk to form ordered higher structures (i.e., fibers) through the formation of silk–CBD dimers [113]. The addition of CNCs to a silk–CBD solution allowed further alignment of the fibers, resulting in the development of a highly ordered and transparent material [113]. The produced films can be utilized in regenerative medicine. This biomaterial would be particularly well suited for cornea regeneration because of its highly transparent nature.

## 6. Conclusions

**Figure 2:**
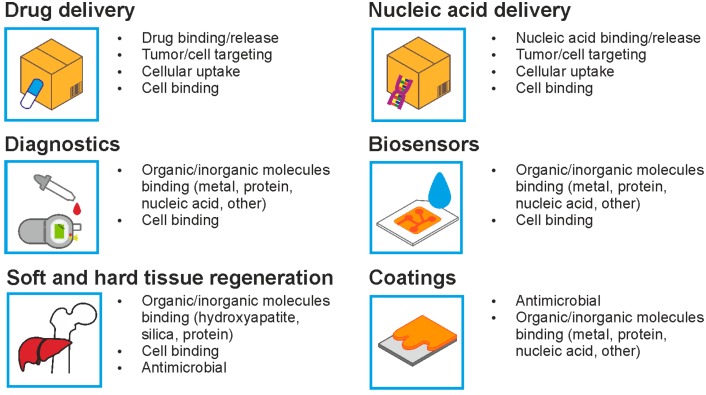
The functionalization of bioengineered silk towards applications.

Among the many proposed biomaterial platforms, silk-based materials stand out because of their extraordinary mechanical properties, biocompatibility and biodegradability. The various modifications that can be applied to silk proteins, either at the DNA level or in the course of further decoration, provide many opportunities to utilize silk in biomedicine (Figure 2). Moreover, the genetic engineering of silk-based materials allows for further improvement of silk’s remarkable properties, generating new silk-like proteins. Currently investigated silk-based materials may soon be applied in cancer treatment [114], in regenerative medicine [115] and as gene-delivery systems [62]. Some silk materials are already being used in clinical trials [116]. Moreover, the database of peptide sequences (PepBank) can be helpful for further development of functionalized silk-based biomaterials [117]. With numerous potential modifications that have yet to be explored and applied to silk, a silk-based polymer is a very promising platform for safe biomaterials with tunable features.

## Figures and Tables

**Table 1:** Functionalization of bioengineered silks with peptides.

Functionalization	Peptide	Bioengineered Silk/Origin	Function of Peptide	Structure	Reference
**Tumor targeting**	CGKRK	6-mer/MaSp1 *N. clavipes*	Targeting tumor vessels	Complexes pDNA/silk	[66]
	F3	1-mer, 6-mer/MaSp1 *N. clavipes*	Targeting nucleolin	Complexes pDNA/silk	[65,66]
	Lyp1	1-mer/MaSp1 *N. clavipes*	Targeting lymphatic vessels	Complexes pDNA/silk	[65]
	H2.1	MS1/MaSp1 *N. clavipes* MS2/MaSp2 *N. clavipes*	Targeting Her2+ receptor	Spheres	[56,72]
	H2.2	MS1/MaSp1 *N. clavipes* MS2/MaSp2 *N. clavipes*	Targeting Her2+ receptor	Spheres	[56,72]
**Cellular uptake**	R_8_G	eADF4(C16)/ADF4 *A. diadematus*	Cell penetrating	Spheres	[51]
	KN	MS2/MaSp2 *N. clavipes*	Cell penetrating	Spheres	[55]
	K_15_	6-mer/MaSp1 *N. clavipes*	Cell penetrating	Complexes pDNA/silk	[62]
	RGD	eADF4(C16)/ADF4 *A. diadematus*	Targeting integrins	Spheres	[51]
		6-mer/MaSp1 *N. clavipes*	Targeting integrins	Complexes pDNA/silk	[64]
	ppTG1	6-mer/MaSp1 *N. clavipes*	Cell penetrating	Complexes pDNA/silk	[54]
	Tat	eADF4(C16)/ADF4 *A. diadematus*	Cell penetrating	Spheres	[51]
**Nucleic acid binding**	K_15_	1-mer, 6-mer/MaSp1 *N. clavipes*	Binding nucleic acids	Complexes pDNA/silk	[62,64,65,66]
	KN	MS2/MaSp2 *N. clavipes*	Binding nucleic acids	Complexes CpG-siRNA/silk, spheres	[55]
**Cell binding**	IKVAV	4RepCT/MaSp1 *E. australis*	Targeting integrins	Fibers, films and foams	[75,76]
	YIGSR	4RepCT/MaSp1 *E. australis*	Targeting integrins	Scaffold	[76]
		Light chain/*B. mori*	Targeting integrins	Films, sponges	[79]
		Heavy chain/*B. mori*	Targeting integrins	Films, sponges	[79]
	RGD	eADF4(C16)/ADF4 *A. diadematus*	Targeting integrins	Films	[77]
		4RepCT/MaSp1 *E. australis*	Targeting integrins	Films	[80]
		4RepCT/MaSp1 *E. australis*	Targeting integrins	Fibers, films, foams	[75,76]
		4RepCT/MaSp1 *E. australis*	Targeting integrins	Coatings, fibers	[6]
		15-mer/MaSp1 *N. clavipes*	Targeting integrins	Fibers, films	[74]
		Heavy chain/*B. mori*	Targeting integrins	Films, sponges	[79]
		Light chain/*B. mori*	Targeting integrins	Films, sponges	[79]
**Anti-microbial**	Mag	4RepCT/MaSp1 *E. australis*	Anti-microbial	Coatings, fibers	[6]
	HNP-2	6-mer/MaSp1 *N. clavipes*	Anti-microbial	Films	[82]
	HNP-4	6-mer/MaSp1 *N. clavipes*	Anti-microbial	Films	[82]
	Hepcidin	6-mer/MaSp1 *N. clavipes*	Anti-microbial	Films	[82,83]
**Inorganic molecules binding**	R5	15-mer/MaSp1 *N. clavipes*	Binding silica	Films, fibers,	[85,87]
		6-mer/MaSp1 *N. clavipes*	Binding silica	Soluble, films	[86]
	A1	6-mer/MaSp1 *N. clavipes*	Binding silica	Soluble, films	[86]
	A3	6-mer/MaSp1 *N. clavipes*	Binding silica	Soluble, films	[86]
	VTK	15-mer/MaSp1 *N. clavipes*	Binding hydroxyapatite	Films	[88]
**Metal binding**	Ag-4	6-mer, 15-mer/MaSp1 *N. clavipes*	Binding silver	Films	[89]
	Ag-P35	6-mer/MaSp1 *N. clavipes*	Binding silver	Films	[89]
	U1	6-mer/MaSp1 *N. clavipes*	Binding uranium	Soluble	[90]
	U2	6-mer/MaSp1 *N. clavipes*	Binding uranium	Soluble	[90]

**Table 2:** Functionalization through incorporation of motifs/domains of proteins of different origin-generation of chimeric proteins.

Functionalization	Motif/Domain	Bioengineered Silk/Origin	Function of Incorporated Motif/Domain	Structure	Reference
**Chimeric biopolymers**	Elastin	(GAGAGS)_6_/*B. mori*	Cell binding, drug binding/release, stimuli responsive material	Hydrogels, particles	[92,93,94,95,96,97,98,99]
	Collagen	(GAGAGS)_n_/*B. mori*	Cell binding	Films, scaffolds	[104]
		Histidine-rich silk/*B. mori*	Stimuli responsive material	Hydrogels	[105]
**Inorganic molecules binding**	BSP	6-mer/MaSp1/*N. clavipes*	Binding hydroxyapatite	Films	[107,109]
	DMP1	15-mer/MaSp1/*N. clavipes*	Binding hydroxyapatite	Films	[106]
**Organic molecules binding**	ABD	4RepCt/MaSp1/*E. australis*	Binding albumin	Fibers, films	[110]
	M4	4RepCt/MaSp1/*E. australis*	Binding biotin	Fibers, films	[110]
	C2	4RepCt/MaSp1/*E. australis*	Binding IgG	Fibers, films	[110]
	Z	4RepCt/MaSp1/*E. australis*	Binding IgG	Fibers, films	[110]
	scFv	4RepCt/MaSp1/*E. australis*	Specific binding of molecules	Fibers	[111]
	CBD	15-mer/MaSp1/*N. clavipes*	Binding cellulose	Films	[113]
**Enzyme**	Xylanase	4RepCt/MaSp1/*E. australis*	Degradation of polysacharides	Fibers, films, foams	[112]
